# First *Toxoplasma gondii* isolate from an aborted foetus of European bison (*Bison bonasus bonasus* L.)

**DOI:** 10.1007/s00436-017-5549-0

**Published:** 2017-07-06

**Authors:** Bożena Moskwa, Justyna Bień, Aleksandra Kornacka, Aleksandra Cybulska, Katarzyna Goździk, Michał K. Krzysiak, Katarina Reiterova, Władysław Cabaj

**Affiliations:** 1 Witold Stefanski Institute of Parasitology, Polish Academy of Sciences, Twarda 51/55, 00-818 Warsaw, Poland; 2 Białowieża National Park, Park Pałacowy 11, 17-230 Białowieża, Poland; 3 Institute of Parasitology of the Slovak Academy of Sciences, Hlinkova 3, 04001 Košice, Slovakia

**Keywords:** *Toxoplasma gondii*, Isolation, Genetic characterization, European bison, Aborted foetus, Białowieża Forest

## Abstract

The study was performed on a male European bison (*Bison bonasus bonasus* L.) foetus spontaneously aborted at the fourth or fifth month of pregnancy in the Białowieża Forest. Serum samples from the foetus and mother revealed the presence of antibodies against *T. gondii* (S/P% = 88% and 75%, respectively). Mobile extracellular tachyzoites were first observed in a *Vero* cell culture, 110 days following inoculation of brain homogenate. PCR amplification with TGR1E1 and TGR1E2 primers confirmed the presence of *T. gondii* DNA, which was classified as Type I by PCR-RFLP genotyping. The sequences of 18S ribosomal RNA (18S rRNA) and 5.8S ribosomal RNA (5.8S rRNA) genes; internal transcribed spacer 1 (ITS1) and internal transcribed spacer 2 (ITS2), obtained from *T. gondii* isolate, have been deposited in GenBank (accession number KX459518.1). This is the first in vitro isolation and molecular identification of *T. gondii* from an aborted European bison foetus. The origin of this protozoan isolate indicates that the species is a significant threat to the European bison conservation program implemented in the Białowieża Forest.

## Introduction

*Toxoplasma gondii* is one of the most important parasites of man and animals. It is a widely prevalent coccidian parasite capable of both sexual and asexual reproduction, and it is this flexibility that enables it to infect any warm-blooded animals as intermediate hosts (Dubey 2010). Transmission of *T. gondii* occurs via the faecal-oral route, through the consumption of infected meat, and by congenital transmission from mother to foetus (Dubey 2010; Guo et al. 2015).

The European bison (*Bison bonasus*) is the largest herbivore, and the heaviest living wild land animal, in Europe. The species is protected both by international and national laws. The species has been assigned endangered status by the IUCN Red List, and has been selected as a priority species under the EU Habitat Directive. The bison population at the Białowieża Forest consists of a group of animals maintained in captivity, and a group of animals living in the wild, and these are conserved within the framework of a European program (Krasiński et al. 2011). The conservation and management of European bison is aimed at increasing the number of animals, continuing the reintroduction process, and preserving the genetic diversity of captive and free herds (Pucek et al. 2004). Such actions might include legal protection in every country, according to its current status on Red Lists or Red Data Books, and the creation of free-ranging populations within the territories of national parks or reserves. The European bison in the Białowieża Forest play an important role in the restitution program and protection of the species.

The European bison and the American bison [*Bison bison*] are related to domestic cattle (*Bos taurus*) and they can interbreed. Both species of bison and cattle are considered resistant to *T. gondii* infection (Dubey 2010). Although *T. gondii* can be transmitted transplacentally in cattle, there is no confirmed report of clinical toxoplasmosis in cattle (Dubey 2010). With respect to *T. gondii* infection in European bison, antibodies to *T. gondii* were found in 24 of 95 (23.5%) of free-ranging bison in Poland (Majewska et al. 2014) and all of four captive European bison living in a zoo in the Czech Republic (Sedlák and Bártová 2006). Very low seropositivity has been reported for the American bison: *T. gondii* antibodies have been found in three (3.1%) of 93 bison from Montana (Dubey 1985), and two (0.8%) of 241 from Alaska (Zarnke et al. 2000). Experimentally, a 1-day-old bison fed oocysts of virulent *T. gondii* became infected but remained asymptomatic (Dubey 1983).

The present paper describes the first isolation and genetic characterization of *T. gondii* from the foetus of a European bison.

## Materials and methods

### Animals and source of samples

During a routine investigation, a European bison was found to have spontaneously aborted a male foetus with a gestational age of 4 to 5 months in the Białowieża Forest, Poland in 2014. The foetus was necropsied in the field by the attending veterinarian, and fresh samples of brain and blood of the foetus, and blood from the cow were sent to our laboratory for diagnosis. After separation, the serum from the cow was stored at −20 °C until analysis for antibodies to *T. gondii* and *N. caninum*.

### Serological examination

The serum sample was analysed for the presence of antibodies to *T. gondii* using a multi-species ID Screen Toxoplasmosis Indirect kit (IDvet, Montpellier) and for *N. caninum* using an ELISA kit (IDEXX Laboratories Inc., Westbrook, ME, USA). The analysis was performed according to the manufacturer’s instructions.

### Parasite isolation

The foetus brain was homogenized in PBS and the homogenate incubated in PBS with 0.25% trypsin at 37 °C for 1 h, with constant shaking. The suspension was filtered through sterilized gauze and centrifuged at 400 *g* for 10 min. The supernatant was then discarded and the pellet washed four times with sterile PBS. The sediment was suspended in approximately 10 ml of PBS and seeded into monolayer *Vero* cell culture flasks.

The *Vero* cell monolayers were cultured at 37 °C with 5% CO_2_, in RPMI 1640 medium (Sigma) supplemented with 1% horse serum, 100 μg/ml penicillin, and 100 μl/ml streptomycin. The culture medium was changed each week. Every 3 or 4 weeks, the *Vero* cells were scraped from the primary flask and transferred to a new culture flask. After detection of the parasites, the cultivation of *Vero* cells was continued and the parasites were passaged every 2 weeks to new culture flask. The tachyzoites from the successfully grown cell culture were frozen at −80 °C for further investigation.

### DNA extraction

Total DNA was isolated from the genomic DNA of the purified tachyzoites cultured in *Vero* cells (Macharey-Nagel, Germany) according to the manufacturer’s instructions. The DNA was eluted in 50 μl of distilled water, and its concentration was determined using a NanoDrop ND 1000 Spectrophotometer (NanoDrop Technologies, USA).

### PCR amplification procedures

#### Amplification with TGR1E1 and TGR1E2 primers

Amplification of the isolated DNA was carried out using standard PCR targeted at the *T. gondii* TGR1E gene region in accordance with the protocol described by Lamoril et al. (1996) with some modifications by Kornacka et al. (2016). The following specific primers were used: TGR1E1 (sense): 5′-ATG GTC CGG CCG GTG TAT GAT ATG CGA T, and TGR1E2 (antisense): 5′-TCC CTA CGT GGT GCC GCA TTG CCT. The final positive PCR product was 191 bp in size.

#### Amplification with Np21 and Np6 primers

The primers Np21 and Np6, based on the NC-5 region specific for *N. caninum*, were used in this study (Yamage et al. 1996).

DNA from Nc-1 *N. caninum* and *T. gondii* (RH strain) tachyzoites were used as positive or negative controls, respectively for the PCR protocol. Amplification products were analysed by electrophoresis through a 1.5% agarose gel stained with Gel Red (Nucleic Acid Gel Stain, Biotium) and visualised under UV light using ChemiDoc (Biorad, USA).

### DNA sequencing and analysis

The sequence analysis was based on a DNA fragment of approximately 1000 bp amplified with primers NC18S and NC28S. The reaction was performed according to Vitale et al. (2013).

The PCR products were purified using the Clean-up Product Purification Kit (A&A Biotechnology, Poland) according to the manufacturer’s instructions, and then ligated into an pGEM-T easy cloning vector (Promega). *Escherichia coli* strain XL-1 Blue MRF electrocompetent cells (Promega) were used for cloning. Positive clones were identified by colony PCR with primers directed against vector sequences outside the multi-cloning site. Clones containing inserts were used for further examination. Positive plasmids were purified using GeneAll Exprep Plasmid SV mini (GeneAll, Korea) according to the manufacturer’s instructions.

The concentration of DNA was measured using a NanoDrop® ND-1000 Spectrophotometer (NanoDrop Technologies, USA). The DNA was then sequenced (Genomed, Poland).

Vector NTI® Advance 10 software (Invitrogen, Scotland) was used to assemble the sequence information from each of the isolated clones. The complete sequences were checked for similarities with published sequences in GenBank using BLAST (http://www.ncbi.nlm.nih.gov/BLAST/).

### Genetic characterization

PCR-RFLP genotyping was performed using ten genetic markers: SAG1, SAG2: (5′-3′SAG2, and alt.SAG2), SAG3, BTUB, GRA6, c22-8, c29-2, L358, PK1 and Apico as described previously (Su et al. 2010). Appropriate positive and negative controls were included in all batches. PCR-RFLP genotyping was conducted by USDA, ARS, Beltsville Maryland, USA.

## Result

Macroscopic examinations of the aborted foetus revealed swelling of the subcutaneous head and trunk tissues. Enlarged, fragile, pale brick-coloured fatty degeneration was observed. Bacterial cultures and attempts of virus isolation have not confirmed the presence of these pathogens.

Serum samples from the foetus and mother revealed the presence of antibodies against *T. gondii* (S/P% = 88% and 75%, respectively). Anti-*N. caninum* antibodies were not detected. Mobile extracellular tachyzoites were first observed in *Vero* cell culture 110 days following inoculation of the brain homogenate from the aborted foetus.

**Fig. 1:**
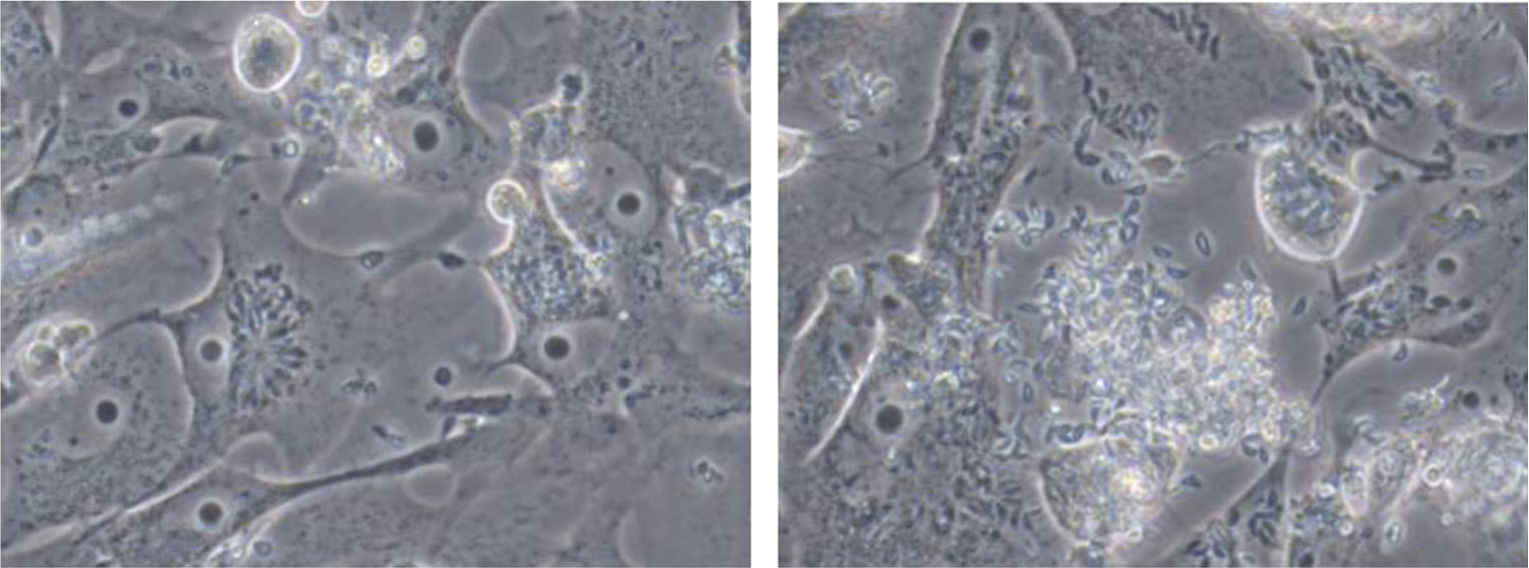
*Toxoplasma gondii* tachyzoites successfully propagated after several passages in *Vero* cell cultures

The tachyzoites were successfully propagated, and many were seen after several passages in cell cultures (Fig. 1).

**Fig. 2:**
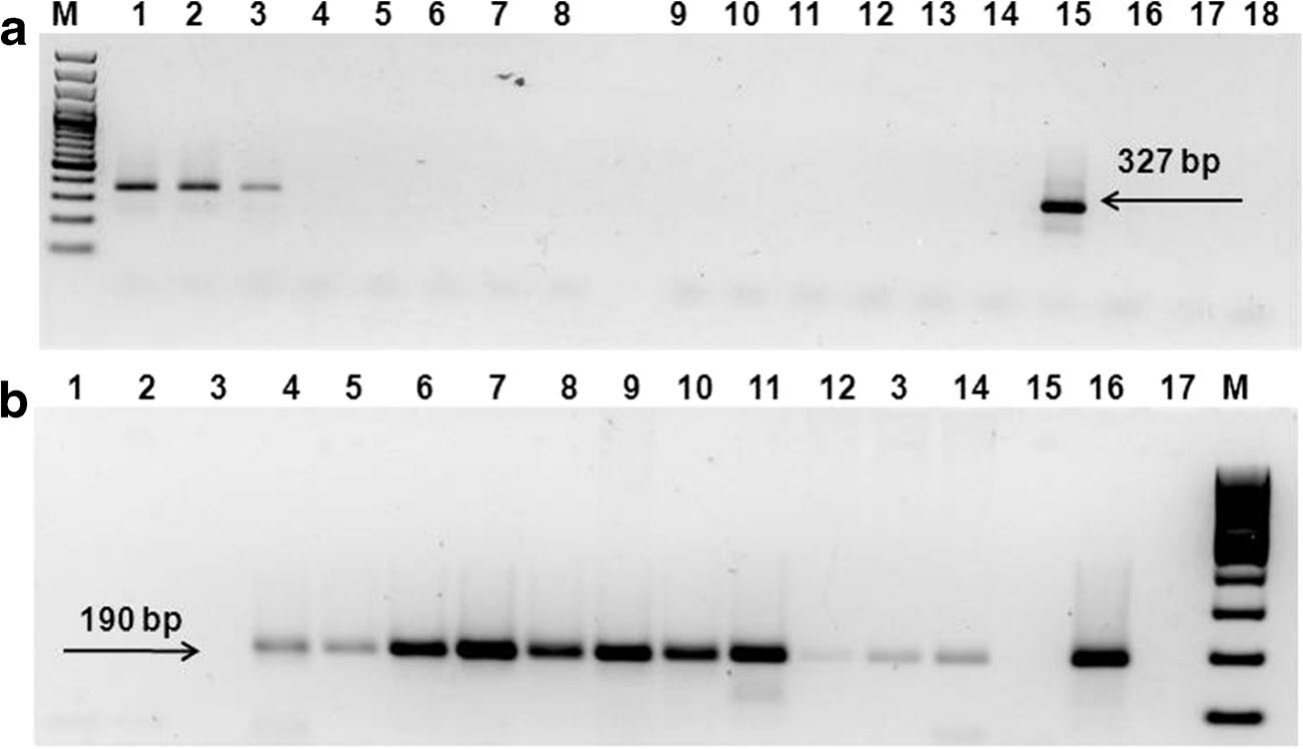
Specific amplicons with primers Np6/Np21 (**a**) and primers TGRE1/TGRE1-2 (**b**)**:** M-molecular marker, lines 1–3—*N. caninum* DNA; lines 4–5—*T. gondii* RH DNA; lines 6–11 – tachyzoites from in vitro culture (*Bison bonasus*); lines 12–14—scrapings (*Vero* cells + tachyzoites); line 15—positive control—*N. caninum* DNA; line 16—positive control—DNA of *T. gondii* RH; lines 17–18—negative control

*T. gondii* DNA was confirmed in the tested samples by the presence of a specific 190 bp amplicon in the examined samples (Fig. 2); however, no 327 bp amplicon confirming the presence of *N. caninum* DNA was detected. PCR-RFLP analysis indicated a Type I genotype for samples 1 and 2 of the *T. gondii* tachyzoites propagated in cell culture (Table 1).

**Table 1:** PCR-RFLP genotyping of *T*. *gondii* isolates from *Bison bonasus*

Strain ID	ToxoDB PCR-RFLP Genotype#	Genetic markers										
		SAG1	(5′ + 3′) SAG2	alt. SAG2	SAG3	BTUB	GRA6	c22-8	c29-2	L358	PK1	Apico
GT-1	#10 (Type I)	I	I	I	I	I	I	I	I	I	I	I
PTG	#1 (Type II)	II	II	II	II	II	II	II	II	II	II	II
CTG	#2 (Type III)	II/III	III	III	III	III	III	III	III	III	III	III
MAS	#17	u-1	I	II	III	III	III	u-1	I	I	III	I
TgCgCa1	#66	I	II	II	III	II	II	II	u-1	I	u-2	I
TgCtBr5	#19	I	III	III	III	III	III	I	I	I	u-1	I
TgCtBr64	#111	I	I	u-1	III	III	III	u-1	I	III	III	I
TgRsCr1	#52	u-1	I	II	III	I	III	u-2	I	I	III	I
Present study												
Sample 1	#10 (Type I)	I	I	I	I	I	I	I	I	I	I	I or III
Sample 2	#10 (Type I)	I	I	I	I	I	I	I	I	I	I	I or III

The DNA sequences of the 18S ribosomal RNA (18S rRNA), 5.8S ribosomal RNA (5.8S rRNA), internal transcribed spacer 1 (ITS1) and internal transcribed spacer 2 (ITS2) genes obtained from *T. gondii* isolate Bb1 (isolate from *Bison bonasus*) have been deposited in GenBank with accession number KX459518.1.

A BLAST-N search using the *T. gondii* isolate Bb1 consensus sequence revealed 99% identity with other *T. gondii* sequences already published in GenBank: *T. gondii* strain YZ-2 (JQ235842.1), *T. gondii* strain YZ-1 (JQ235841.1), *T. gondii* RH (X75429.1) and 97% identity with *T. gondii* strain RH (L25635.1) and *T. gondii* isolate TG-CtPSU49 (KP895862.1).

## Discussion

*Toxoplasma gondii* is a protozoan parasite of both medical and veterinary importance. The species is capable of causing infection and severe disease in both animals and humans. Clinical and subclinical toxoplasmosis has been reported in many host species (Dubey et al. 2007). However, natural *T. gondii* infection does not appear to cause clinical disease or abortion in cattle (Dubey 2010). Infected livestock and game animal species harbouring *T. gondii* tissue cysts represent a risk of infection to human consumers (Guo et al. 2015).

The isolation of *T. gondii* from the brain of the spontaneously aborted foetus confirms that transplacental transmission of the parasite is possible in *Bison bonasus*. However, as no histopathological examination was performed, it remains uncertain whether *T. gondii* was the cause of the abortion.

It has been documented that in Europe and North America the vast majority of *T. gondii* strains could be grouped into three lineages namely Types I, II, III (Howe and Sibley 1995; Dubey 2010). However, statistical analysis indicates significant difference among population in Africa, Asia, Central and South America (Shwab et al. 2014). It is significant that the *T. gondii* isolate in this study was found to be Type I, as these strains are rarely found in nature, perhaps because of their high virulence (Dubey 2010). Types I and II can cause congenital infection in humans, while types II and III are most prevalent in humans and animals in Europe. Interestingly, Type I strains have been isolated from a bovine foetus in Portugal (Canada et al. 2002) and a cow in the USA (Dubey 1992).

Studies have found that European bison are strict herbivores (Pucek et al. 2004; Krasiński et al. 2011). It is possible that the animal in question most likely became infected postnatally by ingesting food or water contaminated with oocysts excreted by felids. The only free-ranging felid in the Białowieża Forest is the Euroasian lynx (*Lynx lynx*), but its population density is not very high (Jędrzejewski et al. 2002). It has been documented that European bison feed on the meadows, pastures and cultivated fields located on the edges of the Białowieża Forest. It is possible that these environments may be contaminated with the faeces of the domestic and wild cats commonly present in this area. The high seroprevalence of *T. gondii* in European bison (25%) (Majewska et al. 2014) indicates the presence of environmental contamination with *T. gondii* oocysts shed by felids, combined with a lack of genetic variation and high level of inbreeding (Dubey 2010; Tokarska et al. 2011). Nothing is known of the prevalence of *T. gondii* in feral domestic and wild felids in Poland and needs investigation.

Toxoplasmosis and other such infections may represent a significant threat for the European bison conservation program (Pucek et al. 2004; Krasiński et al. 2011; Karbowiak et al. 2014). Due to the close relationship of cattle and bison, it is natural to assume that the two host species demonstrate similar clinical symptoms for this infection. Although congenital toxoplasmosis is rarely reported in cattle (Dubey 2010), some examples of reproductive disorders such as spontaneous abortion has been observed as a result of infection (Canada et al. 2002).

## Conclusion

Our study describes the first molecular identification of a *T. gondii* isolate obtained from the aborted foetus of a European bison (*Bison bonasus*), and confirms that it had been acquired by transplacental transmission. Due to the origin of this protozoan isolate, we can assume that the species is a significant threat to the European bison conservation program implemented in the Białowieża Forest. It is important to note that both the aborted foetus and the carcasses of dead animals may represent potential sources of infection for many intermediate and definitive hosts living in this protected area.
